# Dysfunctional mitochondrial processes contribute to energy perturbations in the brain and neuropsychiatric symptoms

**DOI:** 10.3389/fphar.2022.1095923

**Published:** 2023-01-05

**Authors:** Pascal Büttiker, Simon Weissenberger, Tobias Esch, Martin Anders, Jiri Raboch, Radek Ptacek, Richard M. Kream, George B. Stefano

**Affiliations:** ^1^ Department of Psychiatry, First Faculty of Medicine, Charles University and General University Hospital in Prague, Czech Republic, Prague, Czechia; ^2^ Department of Psychology, University of New York in Prague, Czech Republic, Prague, Czechia; ^3^ Institute for Integrative Health Care and Health Promotion, School of Medicine, Witten/Herdecke University, Witten, Germany

**Keywords:** mitochondria, reactive oxygen species, reactive nitrogen species, bipolar disorder, depression, schizophrenia, SARS-CoV-2, HIV-1

## Abstract

Mitochondria are complex endosymbionts that evolved from primordial purple nonsulfur bacteria. The incorporation of bacteria-derived mitochondria facilitates a more efficient and effective production of energy than what could be achieved based on previous processes alone. In this case, endosymbiosis has resulted in the seamless coupling of cytochrome c oxidase and F-ATPase to maximize energy production. However, this mechanism also results in the generation of reactive oxygen species (ROS), a phenomenon that can have both positive and negative ramifications on the host. Recent studies have revealed that neuropsychiatric disorders have a pro-inflammatory component in which ROS is capable of initiating damage and cognitive malfunction. Our current understanding of cognition suggests that it is the product of a neuronal network that consumes a substantial amount of energy. Thus, alterations or perturbations of mitochondrial function may alter not only brain energy supply and metabolite generation, but also thought processes and behavior. Mitochondrial abnormalities and oxidative stress have been implicated in several well-known psychiatric disorders, including schizophrenia (SCZ) and bipolar disorder (BPD). As cognition is highly energy-dependent, we propose that the neuronal pathways underlying maladaptive cognitive processing and psychiatric symptoms are most likely dependent on mitochondrial function, and thus involve brain energy translocation and the accumulation of the byproducts of oxidative stress. We also hypothesize that neuropsychiatric symptoms (e.g., disrupted emotional processing) may represent the vestiges of an ancient masked evolutionary response that can be used by both hosts and pathogens to promote self-repair and proliferation *via* parasitic and/or symbiotic pathways.

## Introduction

Mitochondria represent an endosymbiont model of complex organelle development driven by evolutionary modification of permanently enslaved primordial purple nonsulfur bacteria (Gray et al., 1999). From a teleological perspective, this relationship serves to enhance eukaryotic cellular energy metabolism *via* a convergence of processes within the mitochondrial matrix that optimize the synthesis of adenosine triphosphate (ATP) from adenosine diphosphate (ADP) and inorganic phosphate. Both bacterial and mitochondrial ATP synthases (F-ATPases) require a defined membrane potential to transduce a proton-motive force across the inner membrane resulting in the high-efficiency production of ATP (Gray et al., 1999). To achieve this goal, the bacterial plasma membrane has responded to evolutionary constraints and undergone conversion into the inner mitochondrial membrane. The aforementioned proton-motive force is then functionally coupled *via* a series of chemical events that are facilitated by discrete protein subunits within the transmembrane domains of the F-ATPases, including those that promote sequential protonation and deprotonation of glutamate (GLU) side-chains of cytochrome c-subunits within functional pores. Evolutionary constraints are likely to provide a survival advantage to the host eukaryotic cell and facilitate the optimization of energy production (Stefano et al., 2012). A series of elegant studies published by Watt and others (Watt et al., 2010) confirmed this critical contention in experiments that revealed the enhanced metabolic efficiency of eukaryotic F-ATPases (2.7 versus 3.3–5 protons per ATP molecule synthesized by eukaryotic versus prokaryotic F-ATPases, respectively).

Mechanistically, endosymbiosis has resulted in the apparent seamless coupling of cytochrome c oxidase (COX) to F-ATPase, resulting in maximal ATP production. This has also resulted in the essential partitioning of glycolysis and the tricarboxylic acid (TCA) cycle within specific and discrete cellular domains. COX is a multi-subunit inner mitochondrial membrane enzyme complex, that is, produced and assembled as a mosaic from both nuclear and mitochondrial genes. In a review of this subject, Pierron and others (Pierron et al., 2012) presented evidence suggesting that COX functioned as the critical regulator of mitochondrial ATP production. Specifically, the authors proposed that the addition of nuclear-encoded COX subunits to this process provides the eukaryotic host cell with more effective control over the activity of similar COX subunits encoded by the primordial mitochondrial (mt) DNA genes. This provides the host cell with the capacity to respond more effectively to fluctuating mitochondrial oxygen tensions and potentially dangerous levels of reactive oxygen species (ROS).

Mitochondrial energy processes that “fuel” both evolutionary diversity and cognitive processes yield reactive oxygen species (ROS; O_2_^−^, free radicals, peroxides, and others) as a by-product of their endogenous function. This phenomenon has both positive and negative features. High levels of ROS have been linked to cell death and degeneration, notably in disorders that include type 2 diabetes, Alzheimer’s disease, autism, mood disorders and attention-deficit/hyperactivity disorder (ADHD), among others (Michel et al., 2012). These observations have been expanded in recent studies that demonstrate correlations between mitochondrial dysfunction and various psychiatric disorders (Burtscher et al., 2022; Chen et al., 2022; Umare et al., 2022). In these cases, viruses and bacteria were identified as tightly involved in promoting alterations of mitochondrial and host cellular function *via* hypoxia and immune-mediated upregulation of inflammatory factors and conditions associated with oxidative stress.

In this manuscript, we will examine the emergence of psychiatric symptoms from the perspective of mitochondrial energy production and its aberrancies. It is important to recognize that “cognition,” as currently understood in neural networking terms, has evolved to consume a substantial amount of energy. Thus, any modifications or alterations from baseline mitochondrial function may alter brain energy supply and metabolite generation and be manifested by altered cognition (Stefano and Kream, 2015a; Stefano and Kream, 2015b; Stefano et al., 2019b; Esch et al., 2020).

### Mitochondrial dysregulation, ROS, and neuropsychiatric disorders

Over the years, it has become clear that neuropsychiatric disorders share several specific commonalities, most notably a proinflammatory profile and increased levels of oxidative stress that are also shared with other disorders (Esch et al., 2002; Esch and Stefano, 2002). While the proinflammatory events taking place in specific microenvironments may be contributing to detection and repair processes, these phenomena may also be contributing to several distinct clinical conditions and disorders. The pro-inflammatory state may be a reflection of immune and/or neural mediators that play critical roles in normal intrinsic events such as maintenance or surveillance, but that were recruited at an abnormal time or to an abnormal extent. In this case, these dysregulated proinflammatory events could promote the accumulation of degenerating neurons, activated immune cells (e.g., microglia) (Stefano et al., 1994), and metabolic residues (e.g., protein aggregates, aberrant glycation, and lipid peroxidation). Similarly, proinflammatory states initiated in response to ordinary conditions may not undergo regulated termination, thereby leading to a chronic state of activation and progressive deterioration (Stefano and Scharrer, 1994).

Several beneficial functions have been attributed to mitochondrial-derived oxidative stress, i.e., ROS, including its antimicrobial properties and role in maintaining vascular tone, among others (Wang and Zhao, 2009). Likewise, under homeostatic conditions, nitric oxide (NO) radicals regulate cardiovascular, neural, and immune functions (Atkins and Sweatt, 1999; Hu et al., 2006; Lu et al., 2007). However, when aberrantly expressed ROS can have deleterious effects, including the capacity to damage proteins, denature lipids and membranes, and alter nucleic acid structure (Mehta et al., 2012). Because the brain is a specialized and relatively closed compartment, it is particularly susceptible to ROS-mediated damage. The brain is also very sensitive to the impact of ROS due to its comparatively large need for oxygen; a full 20% of the total body consumption of oxygen is utilized by the brain.

Based on results from our earlier work, we hypothesized that hypoxia serves as a physiological sensory mechanism that alerts mitochondria to the need to re-establish a healthy metabolic state (Stefano and Kream, 2015a). ROS generated in response to hypoxia can initiate damage *via* its capacity to destroy several critical biochemicals (e.g., DNA) and thus induce pathology at the organismic level. In addition to cellular dysfunction, ROS may have a signaling function as it can alert the cell to the onset of hypoxia and facilitate the appropriate response. Based on this scenario, hypoxia-mediated alterations to energy metabolism and production of ROS will decelerate mitochondrial function and provide the time needed to adjust and return to homeostasis. Hence, hypoxia may herald a recuperative period; this period may be prolonged by the generation of ROS. However, an overly-traumatic initial assault may overcome these corrective processes. Life overall is an open-ended process and not a loop; thus protective processes can fail even in response to what could be helpful processes. Thus, this short-lived positive response to ROS may be masked depending on the specific insult and the nature of the resulting pathophysiology.

Furthermore, brain tissue contains relatively high concentrations of polyunsaturated (and thus peroxide-sensitive) fatty acids as well as iron, which may also contribute to the pro-oxidative environment. These factors may contribute to the negative sequelae associated with hemorrhagic stroke. Dopamine and GLU oxidation can occur under ischemic conditions and thus exacerbate a proinflammatory state (Badjatia et al., 2012; Mehta et al., 2012; Chen et al., 2013). Lipid peroxidation also yields toxic compounds (e.g., aldehydes) (McCracken et al., 2000). Other dysfunctional modifications include enhanced endonuclease-mediated DNA fragmentation (Cui et al., 2000).

**FIGURE 1:**
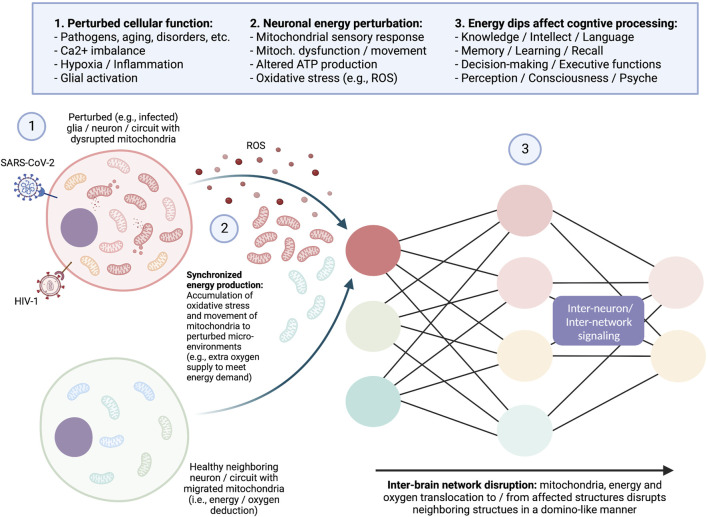
Importance of mitochondrial energy production for functional cognition.

Further cascading pathological pathways resulting from ischemia-associated excitotoxicity and other neurodegenerative insults are triggered by enhanced mitochondrial uptake of calcium by mitochondria and the ensuing synthesis of ROS (Opii et al., 2007; Panov et al., 2007; Hansson et al., 2008; Lemasters et al., 2009). This may occur *via* inhibition of the proton gradient *via* interference with the mitochondrial pore. Because the mitochondrial matrix is negatively charged in this setting, the resulting K^+^ inflow can result in osmotic swelling (Rasola et al., 2010). This cascading series of events will also result in the failure of oxidative phosphorylation and thus result in diminished ATP synthesis. Similar results may emerge in response to virus-induced modulation of cellular Ca^2+^ homeostasis, transcription factor activity (e.g., by human immunodeficiency virus |HIV-1| p300 protein), plasma membrane pumps and channels (e.g., in response to the HIV-1 tat and gp120 proteins), and mitochondrial membrane permeability and potential; these perturbations may result in long-term metabolic alterations (Zhou et al., 2009) (Figure 1).

Hence, numerous studies have described the prevalence of oxidative stress and mitochondrial abnormalities in patients diagnosed with one of several characterized neuropsychiatric disorders (Halliwell, 2006; Arranz and de Leon, 2007; Bouayed et al., 2007; Masood et al., 2008; Tsaluchidu et al., 2008; Bouayed et al., 2009; Marazziti et al., 2012; Tobe, 2013). Specific evidence supporting the role of oxidative stress has been reported in cases of schizophrenia (SCZ), anxiety-related disorders, and mood disorders (Ng et al., 2008; Hovatta et al., 2010). For example, mutations in PTEN-induced kinase 1 (PINK1) may be coupled to oxidative stress and mitochondrial fragmentation (Dagda et al., 2009). In this regard, cGMP-protein kinase G (PKG) signaling pathways (Boess et al., 2004; Werner et al., 2004; Masood et al., 2008), NOX2 processes (Wang et al., 2013) and epigenetic mechanisms (Kano et al., 2013) are factors at play in the pathogenesis of SCZ-related psychiatric disorders. Hence, glutathione deficiency and oxidative stress have been identified as contributing factors to both SCZ and bipolar disorders (BPD) (Kunz et al., 2008; Gigante et al., 2011; Kulak et al., 2013; Wass and Andreazza, 2013) as well as autistic behavior (Rose et al., 2012; Gu et al., 2013). Lipid peroxidation has also been associated with BPD and, somewhat more loosely, with the pathogenesis of SCZ (Versace et al., 2014). Interestingly, oxidative damage associated with NO has also been implicated in the pathogenesis of SCZ and BPD (Wass et al., 2006; Palsson et al., 2009) (Levkovitz et al., 2010). Further implicating mitochondrial dysfunctionality is the finding that electron transport subunits occur at lower levels in these disorders, increasing the presence of ROS (Scola et al., 2013). In both BPD and SCZ higher levels of dopamine are found as well as GLU, chemical messengers which also enhance oxidative stress (Coyle, 2013). Abnormalities associated with the electron transport chain (ETC) system and the mitochondrial complex dysfunctions are also proposed to be involved in autism spectrum disorders (ASD) (Rossignol and Frye, 2012; Gu et al., 2013). Aberrations in pyruvate dehydrogenase activity and copy number variations in mtDNA have been detected in association with autism (Gu et al., 2013). Thus, it appears autism is connected to neuronal dysfunctions associated with metabolic abnormalities in oxidative stress and mitochondrial dysfunction (Ming et al., 2008; Rossignol and Frye, 2012; Frye et al., 2013).

**FIGURE 2:**
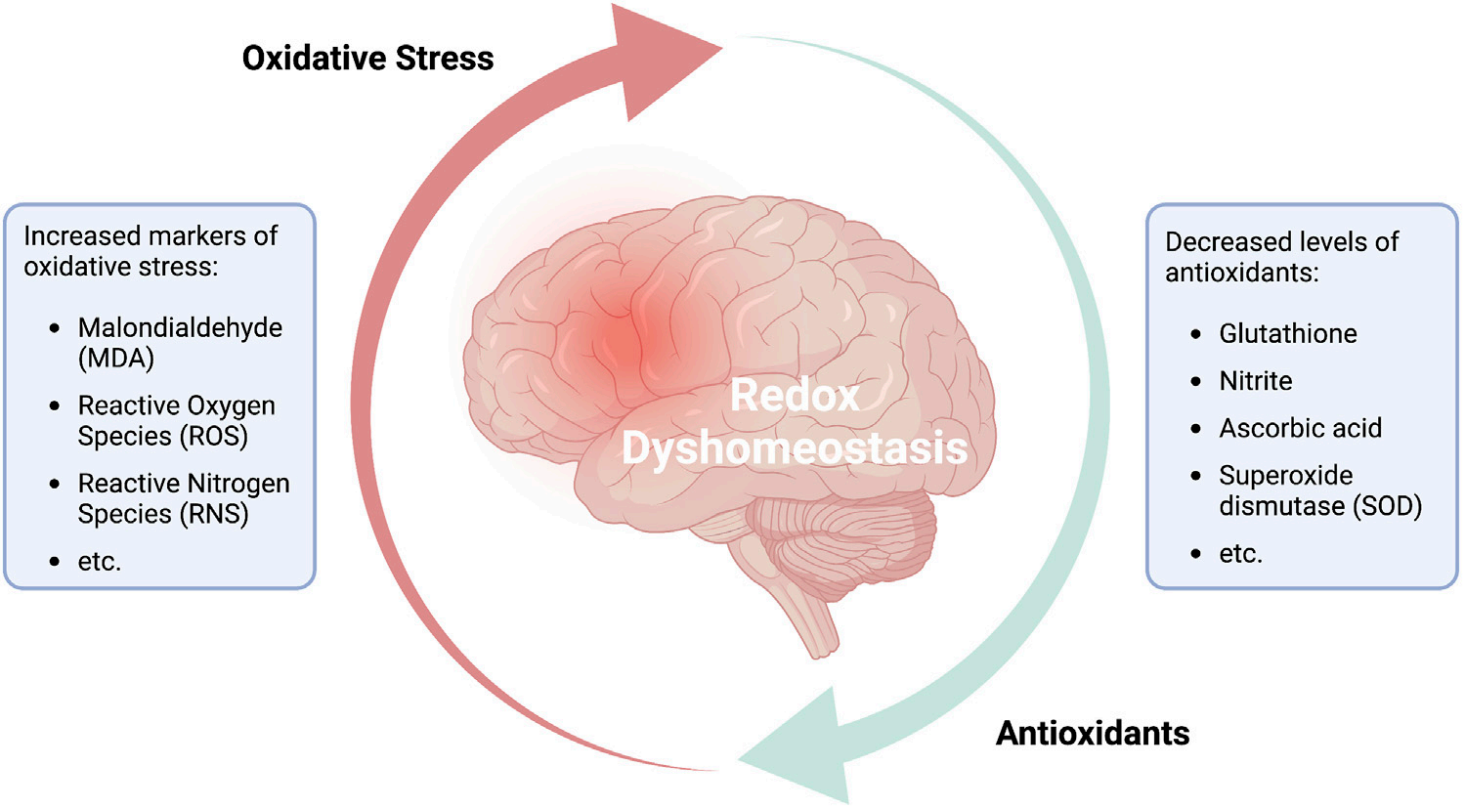
Redox dyshomeostasis: a potential predictor for psychiatric disorders.

The results of a study published by Bajpai et al. (2014) highlighted the close relationship between ROS, mitochondria-energy perturbation, and neuropsychiatric symptoms. The study was conducted on 60 patients diagnosed with unipolar depression and a matching control group of 40 healthy individuals. An analysis of blood samples revealed that the individuals diagnosed with unipolar depression exhibited significantly elevated levels of the lipid peroxidation marker, malondialdehyde (MDA). Production of MDA increases in response to elevated levels of free radicals. Interestingly, serum levels of several critical antioxidants, including nitrite, ascorbic acid, and superoxide dismutase (SOD) were all significantly lower than the levels detected in the control group. Collectively, these results suggested that the oxidant and antioxidant systems were not in balance in patients with unipolar depression. This apparent imbalance may be maintained and exacerbated by psychological stress and associated neuronal inflammation (Figure 2). Similar effects were reported by Lu et al. (2022). In this publication, the authors performed a bidirectional Mendelian randomization analysis of potential causal associations between 11 biomarkers of oxidative stress injury that were evaluated in seven defined psychiatric disorders. The results of this analysis revealed an association of decreased serum levels of ascorbate and bilirubin in individuals diagnosed with ADHD and major depressive disorder (MDD); the authors also noted a nominal association between altered uric acid levels and BPD, ADHD, MDD, SCZ, and anorexia nervosa. Collectively, these results suggest that dysregulated oxidation-antioxidation and a pro-inflammatory environment are linked to the pathogenesis of several common psychiatric disorders. Similarly, results reported by Paternoster et al. (2022) also suggested a link between genetic variation, mitochondrial dysfunction, and psychopathology based on an *in silico* analysis of the impact of disruption of the gene encoding bromodomain containing 1 (BRD1) on mitochondrial function. Interestingly, associations between genetic variation in BRD1 and mental illness have been demonstrated in several genetic studies. Collectively, these findings indicate that modulation of BRD1, which is a known transcriptional regulator of nuclear-encoded mitochondrial proteins, will lead to alterations in mitochondrial physiology, metabolism, and bioenergetics. BRD1-deficiency-associated neuronal changes may have a critical impact on this mechanism and may lead to one or more of the psychopathological manifestations. Finally, results of a study conducted by Baghel and Thakur (2019) revealed that voltage-dependent anion channel 1 (Vdac1), a mitochondrial membrane porin that mediates the transport of energy-associated ions and metabolites (i.e., Ca^2+^, ATP/ADP) has an impact on recognition memory when differentially expressed in the hippocampus. Among their findings, the downregulation of hippocampal Vdac1 in scopolamine-induced amnesic mice as well as its silencing in untreated mice were both associated with mitochondrial disruption, decreased total ATP levels, increased ROS, decreased antioxidant activity, and neuronal degeneration. These findings provide insight into new pathways for brain energy perturbation that have a critical effect on memory, cognition, and potentially mental health.

Hence, the most effective interventions may be those that have a profound impact on mechanisms that modulate acute and chronic inflammation, including those that focus on increasing the level of antioxidants over an extended period (Filiou and Sandi, 2019; Maurya et al., 2022b). Other interventions might target the mitochondria to limit or reduce the impact of a prolonged excited state. Collectively, these findings illustrate that increases in the brain levels of both oxidative and nitrosative stress (reactive nitrogen species [RNS]) combined with a decrease in the antioxidant capacity may be key factors contributing to the etiology of neuropsychiatric disorders and states of consciousness.

### Ancestral factors involved in mitochondrial alteration, cognition, and psychiatry

Results from a series of recent studies strongly suggested that virus-mediated perturbations of mitochondrial function that serve to alter cognition may represent an evolutionarily-conserved response to promote viral survival (Singh et al., 2020; Wang et al., 2020; Stefano et al., 2021; Stefano and Kream, 2022a; Stefano and Kream, 2022b; Buttiker et al., 2022). This targeting hypothesis is based largely on observations focused on the long-term evolutionary co-existence of viruses and prokaryotes, specifically noting that prokaryotic cells have served as an ancient primary target of virus-mediated interactions (e.g., infection with bacteriophages). Given that mitochondria were derived from prokaryotic organisms, this organelle may continue to serve as a prime focus for virus infection. Once the mitochondria have been infected, the virus may then have direct access to the nuclear genome. The intracellular transport of mitochondrial (mt)DNA as nuclear-mitochondrial segments (NUMTs) is an evolutionarily ancient phenomenon (Wei et al., 2022). Of interest, Wei et al. (2022) analyzed whole-genome sequences from 66,083 individuals and found that this transfer process is ongoing and that 90% of these NUMTs underwent transfer comparatively recently in evolutionary terms, i.e., after humans diverged from apes.

Hence, the evolutionary competition between ancestral prokaryotes and viruses based on natural selection and conformational matching of chemical messengers may have been carried forward by mitochondria into eukaryotic cells, emerging most notably in tissues that involve high energy and oxygen demands, such as the brain. As a result, the behavior and function of target tissues (e.g., neuronal cells) may have been subject to alterations that facilitated evasion of the host immune response to material identified as “non-self.” Changes of this nature may have also permitted these cells to become more susceptible to virus infection, replication, and propagation (Stefano et al., 2021). The earliest historical data from human pandemics provided us with a set of intriguing observations, particularly those that identified cognition as a central target even in cases in which the actual causal agent remained unknown. These observations support our view that the brain, most notably the human brain, was and remains an important target because its immune-privileged status permits pathogens to evade the host immunosurveillance (Buttiker et al., 2021b; Stefano, 2021; Buttiker et al., 2022; Stefano, 2021). In our earlier work, we proposed several different neuroinvasive strategies by which viruses (e.g., human immunodeficiency virus 1 [HIV-1], severe acute respiratory syndrome coronavirus 2 [SARS-CoV-2], herpes simplex virus 1 [HSV-1], and Ebola virus, among others) may cross the blood-brain-barrier (BBB). By doing so, these pathogens can exploit the immune privilege of the central nervous system (CNS) to harbor viral genomes. This will permit them to replicate without detection and elicit persistent CNS infection, leading to neuronal dysfunction (Stefano et al., 2022a; Buttiker et al., 2022). Thus, we hypothesize that these viruses may rely on complex signaling pathways and ancient mechanisms of information exchange to hijack the cellular reproductive machinery and alter mitochondrial function. These metabolic disruptions may ultimately lead to many common neuropsychiatric sequelae (Buttiker et al., 2021b; Stefano et al., 2021).

This ancient record of virus-bacteria interactions and its role in promoting altered cognition has been highlighted further by recent findings focused on the increased expression of ancient human endogenous retrovirus type W (HERV-W) envelope (Env) proteins in blood samples from patients diagnosed with psychosis and other neurodevelopmental psychiatric disorders (Balestrieri et al., 2019). Of note, 8% of the sequences found in the human genome originated from HERV infections that occurred millions of years ago (Angrand et al., 2021; Tamouza et al., 2021; Burn et al., 2022). Several groups have reported the increased expression of HERV in SCZ and BPD as well as in various cancers (Diaz-Carballo et al., 2017; Angrand et al., 2021; Tamouza et al., 2021; Burn et al., 2022). In a recent study, Tamouza et al. (2021) detected HERV-W Env proteins in serum samples from subsets of patients with SCZ (−41%) and BPD (−28%), while 96% of the control samples remained negative. These results suggest that the detection of immunoreactive HERV-W Env proteins may be of diagnostic value. Consistent with these findings, Johansson et al. (2020) demonstrated that HERV-W-associated neuropsychiatric symptoms may involve Toll-like receptor 4 (TLR-4) mediated activation of glial cells; the proinflammatory cytokines released in response to this interaction may have an impact on N-methyl-D-aspartate (NMDA)-linked synaptic organization in hippocampal networks and thus synaptic GLU activity.

Activation of immune cells (e.g., microglia, astroglia, and macrophages) and release of inflammatory cytokines can increase synaptic GLU, ROS, and RNS, leading to oxidative stress and a spillover of GLU from synapses into the extrasynaptic space (Haroon et al., 2017). This response may have a negative impact on the number and activity of excitatory amino-acid transporters (EAATs) and lead to further decreases in the astrocyte-mediated clearance of the accumulated GLU. Collectively, these responses will increase the concentration of extrasynaptic GLU, leading to neuronal hyperactivity and excitotoxicity. This effect can be explained at least in part by acute overactivation of synaptic α-amino-3-hydroxy-5-methyl-4-isoxazolepropionic acid (AMPA) and NMDA ionotropic receptors. Prolonged stimulation of presynaptic metabotropic glutamate receptors (e.g., mGluR2/3) and overstimulation of intrasynaptic AMPA receptors may lead to desensitization and loss (Haroon et al., 2017). Hence, over time, acute neuronal overactivity may result in diminished intrasynaptic glutaminergic activity; this may have a direct impact on the function of CNS circuits due to neuronal atrophy and loss. Collectively, these findings suggest that acute and chronic mitochondrial dysfunction, oxidative stress, and aberrant glial function may have significant effects on neuronal communication by altering the synthesis, regulation, and thus the overall balance of excitatory and inhibitory neurotransmitters (Hertz, 2013).

Complementing these studies, Angrand et al. (2021) reported low copy numbers of circulating mtDNA in patients diagnosed with BPD patients at levels that negatively correlated with scales of mood and psychotic disturbance. Diaz-Carballo et al. (2017) reported that cytotoxic stress resulted in the transfer of mitochondria-associated HERV proteins into adjacent cancer cells *via* both tunneling tubes also direct cellular uptake with a considerable translocation (i.e., accumulation) of mitochondria around the nucleus**.** Collectively, these results highlight the multidimensional nature of genetic material and its participation in multiple interactions regardless of its source or origin.

In summary, we hypothesize that, in addition to their expression in the nuclear genome, both viral DNA and mtDNA retain the capacity to communicate with one another and thereby alter cognitive processes (Stefano et al., 2021; Stefano and Kream, 2022a; Stefano et al., 2022b; Stefano and Kream, 2022b; Stefano et al., 2022d). The collective results of numerous published studies suggest that ancient viruses not only remain within the genome, but they can also be activated and expressed in current-day tissues and organisms. However, given their ancient status and their long history of targeting prokaryotic organisms (both bacteria and mitochondria), the viruses emerging today may be poorly-adapted and thus unable to carry out effective interactions with newly-evolved structures associated with human cognitive processes. Hence, the expression of these viruses may result in perturbed cognition. We hypothesize that this response may favor pathogen survival by blunting and/or hijacking one or more of these newly-evolved protective responses, notably those associated with the immediate and ongoing requirements for oxygen-derived energy (i.e., oxidative phosphorylation). Taken together, the dynamic co-evolutionary process that has provided both viruses and bacteria with the opportunity to undergo change/mutation remains under the influence of natural selection. An improved understanding of this relationship will provide us with the means to develop novel therapies that address potentially-related psychiatric disorders and pathologies**,** for example, bacterial “Trojan horses” that carry chemical intervention agents.

### Energy perturbations in the brain and psychiatric symptoms: A cognitive perspective

From a cognitive perspective, mitochondrial dysfunction and oxidative stress may cause perceptual perturbations and neuropsychiatric symptoms *via* brain energy modulation of high-energy demanding neuronal structures (Khacho et al., 2017; Wei et al., 2019; Daniels et al., 2020; Esch et al., 2020; Buttiker et al., 2021b; Picard and Sandi, 2021; Song et al., 2021). This perturbation may be due to intrinsic, for example, epigenetically altered expression of ancient retroviral proteins or various exteroceptive-induced (e.g., immunological) responses and involve the recruitment, translocation, and accumulation of healthy and/or mutated mitochondria (e.g., additional oxygen supply) from surrounding tissues (Wang and Gerdes, 2015; Diaz-Carballo et al., 2017) together with the discussed metabolic dysregulation of mitochondria (i.e., maladaptive molecular interaction) at the site of inflammation (Figure 1). The subsequent shift in brain energy allocation and aberrant local metabolite generation and accumulation (e.g., GLU, ROS, etc.) may cause primary disruption (e.g., hyperactivation, atrophy) of the affected structures (Haroon et al., 2017) and possibly secondary disruption (i.e., energy/oxygen deduction) of other for cognition important high-oxygen demanding systems, such as, for example, the default mode network (DFM). The DFM is a large-scale integrative network responsible for controlling the integration of exteroceptive stimuli and deactivation of external sensory information during resting states, and which interruption is associated with disturbed self-perception and many psychiatric disorders, such as depression, anxiety, and schizophrenia (Xu et al., 2016; Qiao et al., 2017). Hence, acute neuronal inflammation of structures, such as components of the limbic system (e.g., hippocampus), that are essentially involved in memory consolidation and emotional processing can be a powerful promoter of erroneous signal integration into large-scale integrative networks, such as the DFM, creating an energy landscape for maladaptive learning and information processing (Weeber et al., 2002; Angulo et al., 2004; De Chiara et al., 2019).

In a predictive coding model, such disruptions decrease the predictable reliance on affected structures fostering maladaptive forwarding and integration of information into higher-order processing regions of the brain (Buttiker et al., 2021a). If we accept higher function and integrative control networks, such as the DFM, to display naturally high-weighted predictive reliability, their chronic disturbance may not merely lead to temporary disruption of perception, but a persistent modulation of internal concepts and neuropsychiatric sequelae following chronic oxidative stress and mitochondrial dysfunction (Raymond et al., 2017; Buttiker et al., 2021b).

We hypothesize that these mechanisms and ancient evolutionary factors emerged at the same time as cognition and the availability of high-energy producing and high-energy requiring organ systems such as, for example, the CNS. Hence, deeply-embedded primal emotions and associated neuropsychiatric behaviors, including fear-anxiety, sadness-depression, and anger-aggression, among others, may represent a pathway that supports both host-restorative processes and also pathogen replication. Ancient genomic (HERV) and mitochondria sequences become naturally adapted to their microenvironments and thus will ultimately become actively involved in the development of responses to exteroceptive and interoceptive changes and the need to communicate factors associated with different neuronal states. For example, following distressing external/internal events, these sequences may be central to the development of immune activation. The active involvement of both bacteria and viruses in processes associated with brain energy transformation and the generation of neurotransmitters may result in their capacity to “hijack” cellular metabolism. It may also permit bacteria and viruses to use one or more associated predicted constructs (e.g., host behaviors and emotions) to their own advantage. For example, they may develop the capacity to mirror the neuronal conditions associated with depression by using the associated lower energy requirements to mask their own replication. This may deceive the host, who will perceive feelings of depression rather than signs or symptoms of an acute infection. The same situation might be used to explain generalized anxiety and/or psychosis; in this case, hyperactive neuronal structures associated with anxiety symptoms may be used to mask viral activity.

**FIGURE 3:**
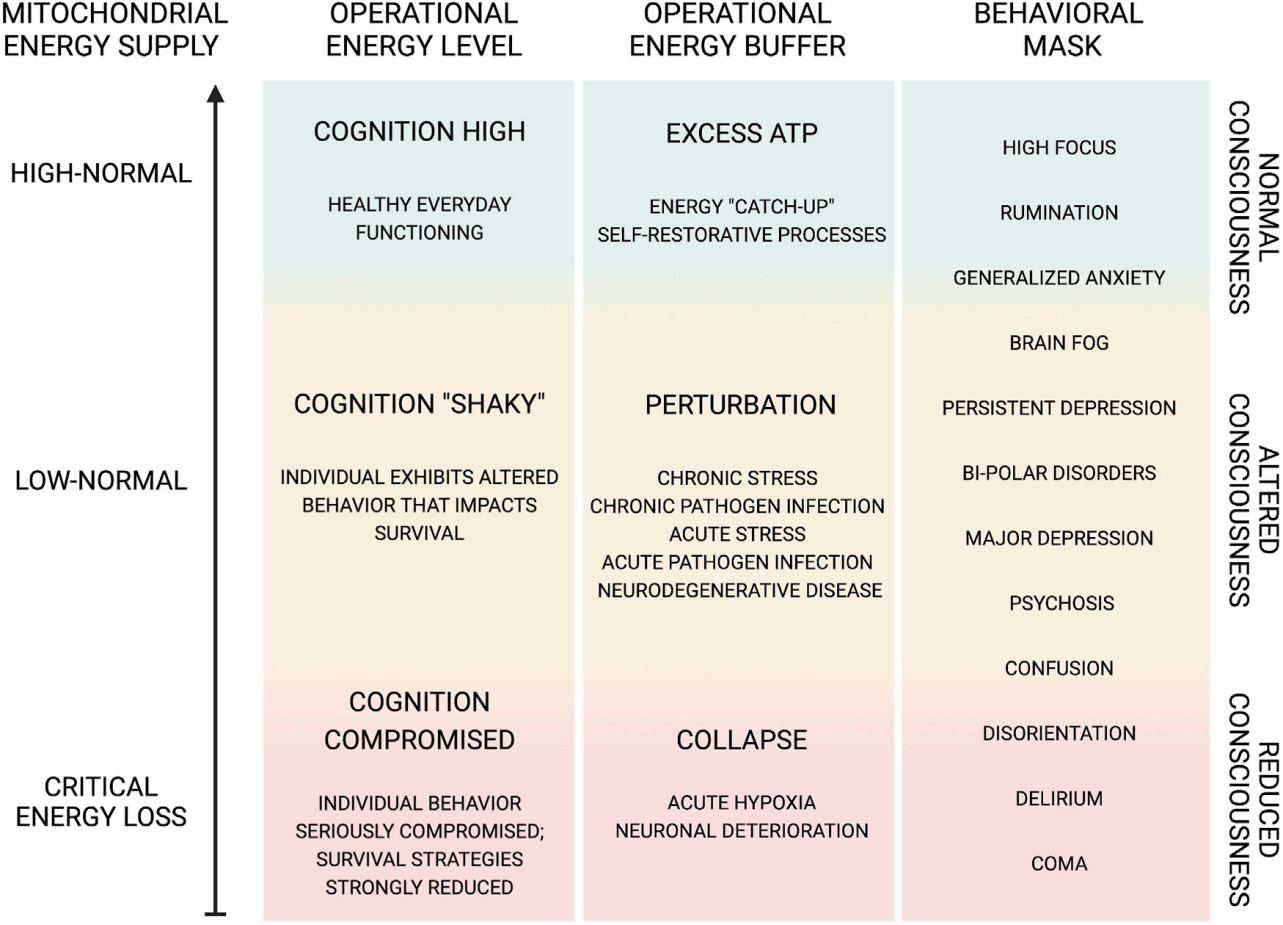
Dynamic functional range of cognition and behavior.

Short-term perturbations may in some cases represent a survival strategy that benefits both the pathogen and the host (Figure 3). As previously discussed, a shift in the allocation of brain energy and generation of metabolites and the resulting alterations in the neuronal microenvironment can induce a temporary change in host perception. This metabolic perturbation may promote pathogen proliferation *via* a symbiotic-parasitic diversion of non-essential neuronal energy from the host while at the same time promoting host protection *via* behavioral adjustments based on external and/or internal circumstances. Examples of this symbiotic-parasitic collaboration might include episodes of depression (e.g., after the loss of a loved one or recovery from a disease or infection, among others), in which physical and mental energy are reduced in the effort to stimulate host recovery; in these cases, the excess energy may be used instead to support pathogen proliferation. Thus, the induction of altered states of consciousness, including confusion, disorientation, and even in some cases anxiety or depression, may serve as positive restorative actions that protect the host. At the same time, some pathogens (e.g., SARS-CoV-2 and HIV-1, among others) may have evolved so that they can induce and/or utilize these cognitive alterations *via* natural selection processes that are sustained and still ongoing as of this day (Stefano et al., 2021; Maurya et al., 2022a; Buttiker et al., 2022; Stefano et al., 2022c).

## Conclusion

As this new energy hypothesis of neuropsychiatric disorders develops, we need to have a better understanding of the endogenous collaboration between mitochondria and host cells in a complex cognitive system. As per this hypothesis, intrinsic biochemical pro-inflammatory factors lead to mitochondrial dysfunction and the generation of ROS. An inflammatory microenvironment may be induced by various internal and external factors or *via* molecular information exchange with pathogens, such as viruses. Recent studies have revealed that many disorders have a pro-inflammatory profile in which oxidative stress is capable of initiating damage and cognitive malfunction. For example, mitochondrial abnormalities and oxidative stress have been implicated in several well-known psychiatric disorders, including mood disorders, SCZ and BPD. Given their role as the powerhouse of the cell, it is reasonable to assume that mitochondria play a significant role in maintaining cognition. Hence, shifts in brain energy supply and/or availability, activation of pro-inflammatory mechanisms, generation of ROS, and release of cytotoxic molecules (e.g., GLU), may result in mitochondrial translocation and dysfunction. These mechanisms may lead to maladaptive signal transduction and information processing and result in neuropsychiatric symptoms. In this manuscript, we highlighted the unique evolutionary position of bacteria-derived mitochondria and the mitochondrial communication pathways that highlight their ancestral co-existence and interactions with viruses. Hence, we hypothesize that these pathogens may have adapted to make use of both mitochondrial processes and associated neuropsychiatric symptoms to mask their presence and promote their continuing proliferation.

Ongoing computational, genetic and neurobiological research into the mechanisms underlying mitochondrial dysfunction, abnormal metabolite generation, and the involvement of those metabolites in neuronal interaction and communication will add more information to our understanding of these phenomena. The collection of additional quantitative findings focused on brain energy distribution in health and disease as well as the continued study of the impact of energy perturbation in specified cortices and neuronal circuits will help to elucidate the contributions of these processes to common neuropsychiatric symptoms. A combined understanding of the contributions of genetic variation and cellular/mitochondrial dysfunction, as well as their impact on energy perturbation in single neurons, neuronal circuits, and brain networks, may provide us with insight into new therapeutic targets that can be used to treat mental illness.

Illustration demonstrating the importance of mitochondrial energy production for the multi-characteristic cognitive ability of the brain as it translates into a mind. This prokaryotic evolved organelle also exhibits sentinel and independent behaviors transcending its energy symbiotic relationship with the host cell. In this regard, hypoxia may be the key signaling event triggering rapid and prolonged influences on this organelle (Esch and Stefano, 2002; Stefano and Kream, 2015b). Thus, pathogens, such as SARS-CoV-2, HIV-1, etc., ROS, proinflammatory stimuli, and perturbations of oxidative phosphorylation will not only impact mitochondria but processes that depend on its immediate ATP production and delivery. The energy-correlated processes are synchronized and sensitive to their microenvironmental surrounding as well as having the ability to be a mobile energy-responsive system between cells and tissues (Stefano et al., 2019b; Stefano et al., 2019a). As such, mitochondria-induced alterations in energy production and translocation from/to affected sites may disrupt communication with neighboring neuronal structures in a domino-like manner. Thus, it is not surprising, given this level of complexity involving numerous chemical messengers and networks, that abnormal cognitive and non-cognitive behaviors may emerge if the system became dysfunctional, even over a short-term perturbation. Further, it can be surmised that acute short-term perturbations, may, in some cases, represent a survival strategy allowing for momentary pausing of non-essential behavioral exhibition, e.g., depression over the loss of a loved one or resting following pathogen infections.

The figure illustrates the continuous cycle of reduction oxidation (redox) reactions, which are central for the functionality of every bodily cell. The fine-tuned interplay between the generation of free radicals and their reduction through antioxidant activity has been established a fundamental part in natural aging, health and disease. An imbalance between markers of oxidative stress and levels of antioxidants in the brain is associated with mitochondrial dysfunction and neuronal inflammation and has been demonstrated in various brain disorders and psychiatric disorders. Although the structure and valence of redox dyshomeostasis may vary among the different pathologies, it has been confirmed that various depressive and behavioral disorders show decreased levels of antioxidants and related enzymes (e.g., ascorbate, glutathione, SOD, etc.) and heightened levels of peroxidation markers and free radicals (e.g., MDA, ROS/RNS, etc.). Hence, redox dyshomeostasis may prove as a reliable marker for identifying psychiatric disorders or determining the danger in acquiring such. In addition, understanding the redox structures and deviations from the healthy norm, may allow the development and utilization of customized medicinal interventions.

Illustration of the dynamic function capacity and range of cognitive functions, all of which are dependent on a bountiful energy supply and their behavioral significance. Cognitive function can exhibit modified behavioral output due to various factors, such as oxygen and glucose supply as well as pathogen-initiated perturbations either directly or indirectly affecting mitochondrial oxidative phosphorylation (Khacho et al., 2017; Daniels et al., 2020). The altered cognitive state, e.g., confusion, disorientation, etc., may be involved in positive restorative actions protecting the host. We further speculate that pathogens, e.g., SARS-CoV-2, HIV-1, etc., may induce cognitive alterations as an intentional evolutionary action, arising from natural selection, which is sustained and refined today (Stefano et al., 2021; Buttiker et al., 2022; Stefano et al., 2022d). Thus, pathogen-induced cognitive alterations may benefit the reproductive capacity of neuronal viruses and other CNS pathogens. Indeed, neuronal cell mitochondria become compromised after the integration of the viral genome (Wei et al., 2022). The resulting cognitive impairment benefits viral spread, as infected individuals exhibit behaviors that reduce host protection against infection (Stefano et al., 2021). Pathogens in the CNS may gain entrance by taking advantage of immune cell trafficking, bringing into question how immune-privileged the CNS really is (Stefano et al., 2022a). Furthermore, by hijacking components of a host cell, e.g., mitochondria, pathogens may be laying the ground for a re-infectious event, including altered cognition. Again, HIV-1 and SARS-CoV-2 may give rise to persistent pathological actions (Buttiker et al., 2022). In this regard, we further speculate, in part, idiopathic cognitive and/or other neuropathological and neuropsychiatric disorders may be representative of previous pathogen infections whose origins and presence have remained hidden or masked. Hence, we suggest that disorders, such as depression, anxiety, etc., do not merely represent perturbations of brain energy states, but these symptoms may be used to buffer and reallocate energy supply for self-restorative or pathogen proliferative purposes. If the extent of this phenomenon is born out, the prospect of medicinally treating these disorders either with typical pharmaceutical agents or novel ones, e.g., protease inhibitors (Stefano et al., 2022a), designed to take advantage of the viral cell trafficking and mitochondrial targeting routes of infection, will gain in significance.
